# Using Long-Duration Static Stretch Training to Counteract Strength and Flexibility Deficits in Moderately Trained Participants

**DOI:** 10.3390/ijerph192013254

**Published:** 2022-10-14

**Authors:** Konstantin Warneke, Lars H. Lohmann, Michael Keiner, Carl-M. Wagner, Tobias Schmidt, Klaus Wirth, Astrid Zech, Stephan Schiemann, David Behm

**Affiliations:** 1 Department for Exercise, Sport and Health, Leuphana University, 21335 Lüneburg, Germany; 2 Institute of Sport Science, Carl von Ossietzky University Oldenburg, 26129 Oldenburg, Germany; 3 Department of Training Science, German University of Health & Sport, 85737 Ismaning, Germany; 4 Department Training and Testing Science, Medical School Hamburg, 20457 Hamburg, Germany; 5 Sport and Exercise Sciences, University of Applied Sciences, 2700 Wiener Neustadt, Austria; 6 Department of Human Motion Science and Exercise Physiology, Friedrich Schiller University, 07743 Jena, Germany; 7 School of Human Kinetics and Recreation, Memorial University of Newfoundland, St. John’s, NL A1C 5S7, Canada

**Keywords:** muscular imbalance, maximal strength, long-lasting stretching, flexibility, maximal voluntary contraction, rehabilitation

## Abstract

Many sports injuries result in surgery and prolonged periods of immobilization, which may lead to significant atrophy accompanied by loss of maximal strength and range of motion and, therefore, a weak-leg/strong-leg ratio (as an imbalance index ∆ ) lower than 1. Consequently, there are common rehabilitation programs that aim to enhance maximal strength, muscle thickness and flexibility; however, the literature demonstrates existing strength imbalances after weeks of rehabilitation. Since no study has previously been conducted to investigate the effects of long-duration static stretch training to treat muscular imbalances, the present research aims to determine the possibility of counteracting imbalances in maximal strength and range of motion. Thirty-nine athletic participants with significant calf muscle imbalances in maximal strength and range of motion were divided into an intervention group (one-hour daily plantar flexors static stretching of the weaker leg for six weeks) and a control group to evaluate the effects on maximal strength and range of motion with extended and bent knee joint. Results show significant increases in maximal strength (d = 0.84–1.61, *p* < 0.001–0.005) and range of motion (d = 0.92–1.49, *p* < 0.001–0.002) following six weeks of static stretching. Group * time effects (*p* < 0.001–0.004, η² = 0.22–0.55) revealed ∆ changes in the intervention group from 0.87 to 1.03 for maximal strength and from 0.92 to 1.11 in range of motion. The results provide evidence for the use of six weeks of daily, one hour stretching to counteract muscular imbalances. Related research in clinical settings after surgery is suggested.

## 1. Introduction

Maximal strength (MSt) is defined as the capability to perform a maximal voluntary contraction (MVC) against an external resistance [1] and is perceived as a key determinant of athletic performance [2,3] as well as injury prevention [4] and rehabilitation [5]. Baar [6] highlighted that musculoskeletal injuries account for more than 70% of cases where athletes refrain from competing or training for their sport. Consequently, many athletes will not be able to perform at their pre-injury level [7,8], and the return to sport requires a symptom- and pain-free status [9].

Therefore, there is a need for effective and time-efficient rehabilitation programs that can be implemented as soon as possible to minimize an athlete’s time away from training and competition [10]. Although early mobilization was found to be beneficial [11], and commonly implemented rehabilitation programs use physiotherapy and mobilization, they generally fail to fully restore MSt and muscle thickness (MTh). Stevens et al. [12] found a persistent 20% difference in plantar flexor strength, while Vandenborne et al. [13] stated a persistent 5.5% deficit in muscle cross-sectional area (MCSA) after ten weeks of a regular rehabilitation program in previously injured and immobilized subjects. Davies and Sargeant [14] found an 11% deficit in muscle volume after 50 days of exercise therapy, which was later confirmed by Ingemann-Hansen and Halkjaer-Kristensen [15]. Interestingly, Lantto et al. [16] showed that even 11 years after an Achilles tendon rupture, subjects demonstrated a 5% calf muscle peak torque deficit. Based on these reports, it can be assumed that time-extended and more comprehensive or intensive rehabilitation is required to fully restore muscular strength following injury and thus reduce muscular imbalances [17].

Generally, muscular imbalances can be seen as a risk factor in sports [18] as they alter the kinetics of different movements. Therefore, incomplete recovery of MSt and range of motion (ROM) seems to influence the subsequent injury history of athletes leading to problems in their future careers. The risk of hamstring strain injuries seems to increase with higher level strength imbalances (e.g., knee flexor weakness, bilateral knee flexor strength asymmetry, hamstrings to quadriceps ratios), higher levels of fatigue and lower levels of flexibility [19,20]. Similarly, Croisier et al. [18] reported significantly higher injury risks for soccer players that exhibited strength imbalances in their lower limbs in pre-season testing, which was confirmed by Fousekis et al. [21] pointing out a higher risk of muscle strains due to strength and muscle length imbalances in soccer players. Gabbett [22] showed that about 17.9% of injuries in professional rugby players can be attributed to the calves and thighs. In soccer, up to 52.8% of all injuries among high school athletes were reported for the lower extremities [23,24]. Ankles (40%), knees (25%) and thighs (14%) are especially prone to high injury rates, whereby sprains (50%) and strains (17%) were the most common diagnoses.

Accordingly, especially in (elite) sports, sufficient muscular balance and high levels of MSt seem to be of great importance to increase performance and facilitate a rapid rehabilitation to be able to return to training and competition as Croisier [25] emphasized the enhanced risk of injury due to ROM and MSt lower limb imbalances. Consequently, there is a need to implement early and effective training routines in the rehabilitation of unilateral injuries that lead to immobilization-induced strength deficits in one leg. If the aim is to increase MSt, strength training is commonly used. Suetta et al. [26] demonstrated that post-operative resistance training increased MSt and muscle function to a larger extent when compared to common rehabilitation regimes showing effect sizes of 0.86–0.97, dependent on weekly training frequency [27]. Especially in older individuals, but also in the general population, there is a high request for safe and efficient training programs in rehabilitation and prevention [28], in particular, because commonly used rehabilitation programs seem to not be sufficient to restore the functionality of previously injured tissue [12,16]. However, considering the assumption that, especially directly after surgery, a strength training must not be performed, it seems beneficial to resort to alternative training routines to accelerate the rehabilitation process. Results from animal studies provide evidence that long-duration stretch training also leads to significant increases in MSt, MTh and muscle length [29] with an assumed transferability to humans [30,31]. As static stretching does not require active (joint) movement (i.e., reduced activation of the central nervous system) and can be included in seated activities, it can be performed with a high frequency (i.e., daily). It can be hypothesized that the inclusion of static stretching in rehabilitation programs is beneficial to improve the rehabilitation outcome. Based on this premise, the present study aims to examine the effects of long-duration static stretch training on calf muscles with persistent MSt imbalances on plantar flexors MSt and ROM.

## 2. Materials and Methods

### 2.1. Study Design

This study was performed as a single-center, pragmatic controlled trial. Participants were divided into a static stretch training group (IG) and a non-intervened control group (CG). Since the intervention, as well as the testing procedure, was performed unilaterally, the statistical calculation was performed for the intervened leg (IL) and the non-intervened control leg (CL) in IG as well as for the strong leg (CGs) and the weak leg (CGw) of the control group to assess the effects of daily stretching training for six weeks in each leg. Maximum isometric strength (MISt) and ankle dorsiflexion range of motion (ROM) with flexed and extended knee joints were examined at pre- and post-test. Before testing, a warm-up routine was performed that consisted of five minutes of bodyweight ergometer cycling with 1 Watt/kg.

### 2.2. Participants

**Figure 1:**
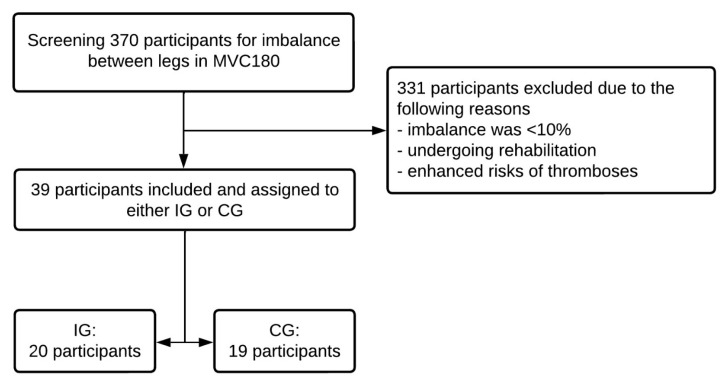
Flow chart illustrating inclusion for the study.

Three hundred and seventy (370) participants were initially recruited from North-West Germany (Oldenburg, Germany) between September 2021 and April 2022 from a sports study program and local sports club. To answer the research question, MSt values of the plantar flexors were screened for imbalances of at least 10% between legs, as Chin et al. [32] refer to enhanced risk of injury due to a 10% bilateral leg strength difference and Lantto et al. [16] and Stevens et al. [12] demonstrated muscular imbalances after finishing rehabilitation. Participants that demonstrated an MSt difference of at least 10% between their right and left plantar flexors in the extended knee MVC testing in the pre-test were included for further evaluation. A total of 331 subjects were excluded because of a strength imbalance of less than 10%, undergoing rehabilitation programs or exhibiting enhanced risks of thromboses at the time of the study. Consequently, 39 male, athletically active participants (age: 28.7 ± 3.2 years, height: 179.9 ± 5.2 cm, weight: 83.6 ± 6.3 kg) were included for further calculation (see Figure 1). Participants were allocated to IG and CG based on their willingness to perform a daily one-hour static stretching protocol: IG comprised 20 and CG 19 participants. The origin of the muscular imbalances varied between participants and was not exclusively attributed to previous injuries. All participants performed the required stretching frequency and duration, and thus, effects were calculated considering all values without any dropout.

All participants were informed about the experimental risks and provided written informed consent to participate in the present study. Furthermore, approval for this study was obtained from the institutional review board (Carl von Ossietzky University Oldenburg, No.121-2021). The study was performed in accordance with the Helsinki Declaration. 

### 2.3. Testing Procedure

#### 2.3.1. Maximal Strength Testing

**Figure 2:**
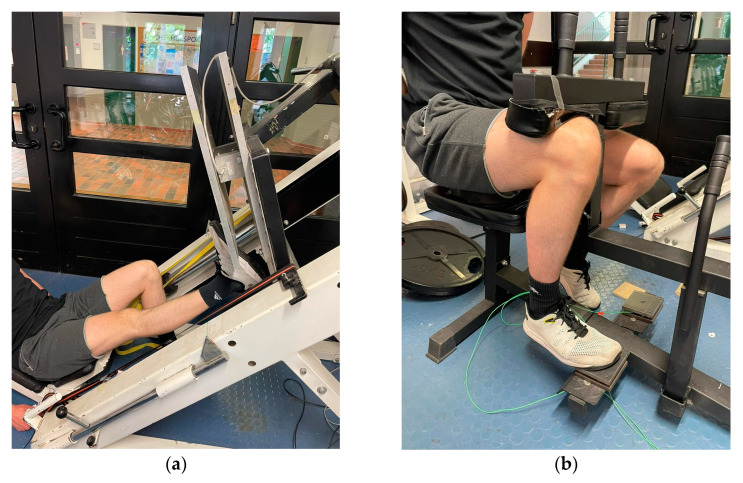
Measurement of MSt in the extended knee joint (**a**) and the flexed knee joint (**b**).

MSt in the plantar flexors was determined via isometric MVC in extended (180° knee joint) (MVC180) and flexed (90° knee joint) (MVC90) knee joints using single-leg testing for both legs separately. For isometric strength measurements, a high reliability (ICC = 0.95) can be assumed [31]. MVC180 was examined via a 50 × 60 cm force plate and a force transducer (company AST, Leipzig, model KAC) with a measurement range of ±5000 N and a 13-bit analog-to-digital converter attached to the sled of a 45° leg press. The leg press sled was fixed in position with industrial-grade tensioning straps so that the starting position was set to an ankle joint angle of 90° with the metatarsophalangeal joint of the foot placed on the edge flush (see Figure 2a). The testing procedure and devices were validated and described in [30,31]. Subjects performed an isometric MVC against the force plate in response to an acoustic signal and held the contraction for three seconds. Subjects rested for one minute between repetitions to avoid fatigue. Measurements were conducted until no improvement in MISt was recorded with a minimum of three trials. MVC90 was tested using a calf muscle testing device (CMD) equipped with 10 × 10 cm force plates attached to the footrests and force sensors “Kistler Element 9251” with a resolution of 1.25 N, a pull-in frequency of 1000 Hertz and a measurement range of ±5000 N. A charge amplifier Type5009 and a 13-bit analog-to-digital converter NI6009 as used to record the vertical forces (Fz). The thigh pad was fixed in position using industrial-grade tensioning straps so that the starting position was set to an ankle joint angle of 90° with the metatarsophalangeal joint of the foot placed on the edge flush (see Figure 2b). Subjects performed an isometric MVC in plantar flexion for three seconds in response to an acoustic signal. Testing was performed until the subject could not improve the achieved MSt values with a minimum of three trials. The reliability of the MSt testing procedure is stated in Table 1.

#### 2.3.2. ROM Measurement

**Figure 3:**
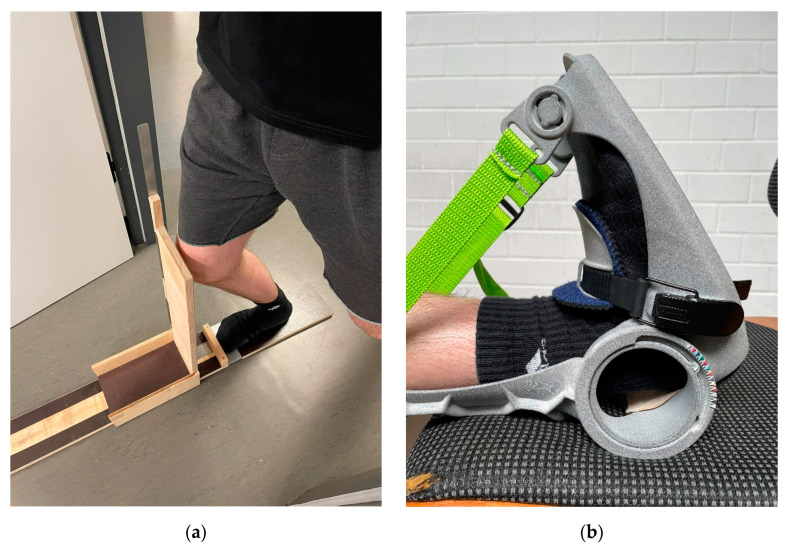
Measurement of ROM via KtW (**a**) and ORTH (**b**).

Upper ankle joint ROM was investigated using the “knee-to-wall stretch” test (KtW) and the goniometer on the orthosis (ORTH) in both legs. A sliding device was utilized to measure the ROM in the upper ankle with a bent knee joint via KtW (see Figure 3a), as previously described in [31]. Subjects were instructed to place the foot to be tested on the attached marker of the device (see Figure 3a). They were instructed to keep the contralateral leg off the floor and to stabilize the body with their hands on a door frame. Subjects pushed the board of the sliding device forward with their knee until the heel of the standing leg started to lift off. During this procedure, one investigator constantly pulled on a sheet of paper. Once the paper could be pulled from underneath the heel, the measurement was stopped. The distance the sliding board moved forward was read off in cm from the attached measuring tape. Participants had to perform three valid trials per leg. ROM assessment with this method can be classified as high with ICC = 0.98–0.99 [31]. ROM in dorsiflexion with an extended knee joint was measured via ORTH (see Figure 3b). For this purpose, the foot of the participant was placed on an object the same height as the chair. The foot was fixed in the orthosis and moved into a maximally dorsiflexed position while keeping the knee extended. Testing started from a neutral 0° position in the ankle. Each big indentation of the goniometer corresponds to an increase of 5° and each little indentation to an increase of 2.5°. ROM assessments in the ankle joint using the goniometer of the orthosis can be classified as high with ICC = 0.99 [31]. The reliability of the ROM measurement is provided in Table 1.

### 2.4. Intervention

#### Static Stretch Training

Static stretch was performed as previously described in Warneke et al. [31]. The intervention group (IG) stretched the plantar flexors of their weak leg for one hour per day for six weeks (see Figure 3b). Subjects had to stretch with the use of the orthosis with an extended knee joint. The stretch intensity over the time course of the intervention was documented by the daily achieved ankle angle, which the subjects read from the goniometer in the maximally dorsiflexed position. The subjects were instructed to reach an individual stretching pain of 8–10 on a visual analog scale of 1 to 10 (with 0 = no pain; 10 = unbearable pain). The subjects sat with their backs as straight as possible against the backrest of a chair with their foot in the orthosis on a support object of the same height to ensure the extension of the knee and optimize stretch of the plantar flexors. The participants were instructed to use a stretch diary and fill in the set angle of the orthosis as well as the stretching duration per day. For a more detailed description of the orthosis, see previous investigations that used the orthosis [31]. If participants missed more than one day per week of the stretching, they were excluded for further calculation of effects. The control group did not perform any intervention within the scope of this study (e.g., any form of long-lasting stretching). Subjects in the control group were offered the stretching training after completion of the intervention phase in the intervened group. However, they rejected the offer.

### 2.5. Data Analysis

The data analysis was performed with SPSS 28. Data are provided using Mean (M) ± Standard Deviation (SD). The normal distribution was checked via the Shapiro–Wilk test. The best performances in each test were used for the statistical analysis. Reliability was determined and is provided as intraclass correlation coefficient (ICC), 95% confidence interval (95% CI) and coefficient of variability (CV) for listed monitoring assessments, which are listed in Table 1. Moreover, the Levene test for homogeneity in variance was performed. One-way ANOVA was used to rule out significant differences between groups (and legs) in pre-test values. Two-way ANOVA (two groups × two times) with repeated measures was performed for the collected parameters. The Scheffé test was used as a post-hoc test for mean differences. Effect sizes are presented as Eta squares (η²) and categorized as: small effect η² < 0.06, medium effect η² = 0.06–0.14, large effect η² > 0.14 [33]. Additionally, Cohen’s d [33] effect sizes are reported and categorized as: trivial d < 0.2, small d < 0.5, medium d = 0.5–0.8, and large effects d > 0.8. Furthermore, differences in pre-test values within IG and CG between the legs of the participants were evaluated using a paired t-test, assuming that the legs of a person cannot be viewed to be independent of each other. To investigate the influence of the intervention on muscular imbalances, a ratio between the weak leg and the strong leg was calculated compared to the known hamstrings-to-quadriceps ratio from the literature [34,35]. T Post-hoc power (1-β) was calculated via G-Power (Version 3.1, Düsseldorf, Germany). The level of significance was *p* < 0.05. Pearson correlations were calculated for pre- and post-comparisons in MSt.

## 3. Results

The mean stretching time per week was 6.7 ± 0.1 h per week. The evaluation of the intervened leg (IL), control leg (CL), weak leg of the control group (CGw) and strong leg of the control group (CGs) revealed a significant difference in pre-test values with *p* = 0.04 between the control leg of the intervention group and the weak leg of the control group for MVC180 (the CL of the intervened group was stronger than the weak leg of the CG). No further difference in pre-test values could be observed with *p* = 0.258–0.807. Table 2 shows the descriptive statistics for MVC180, MVC90, KtW and ORTH and the results of two-way ANOVA with repeated measures. 

### 3.1. Effects of Stretching Intervention on MSt

For MVC180, there were time-dependent effects (η² = 0.31, *p* < 0.001) and time x group interactions (η² = 0.52, *p* < 0.001). The Scheffé test showed significantly large magnitude increases from pre- to post-test values in IL and CL (*p* < 0.001, d = 1.46), IL and CGw (*p* < 0.001, d = 1.27) and between IL and CGs (*p* < 0.001, d = 1.61). No significant differences with the other groups could be determined, *p* = 0.51–0.91) (Table 2). Consequently, there was an increase of 19.9% in MVC180 due to the intervention, while no significant increase could be obtained in the control conditions. 

For MVC90, moderate to high magnitude improvements were evident for the time-dependent effect (η² = 0.13, *p* < 0.001) and time x group interaction (η² = 0.2, *p* < 0.001), with IL exceeding the CG (*p* = 0.005, d = 0.84). The Scheffé test determined no significant differences between pre- and post-test differences in IL and CL (*p* = 0.12) and in CL and CGs (*p* = 0.135) (Table 2). Consequently, there was an increase of 9.6% in MVC190, while no significant increases were obtained in the control conditions.

### 3.2. Effects of Stretching Intervention on ROM

For KtW, the two-way ANOVA revealed high effects for the time-dependent effect (η² = 0.11, *p* = 0.003) and the time * group interaction (η² = 0.92, *p* < 0.001). The Scheffé test determined significant, large magnitude improvements from pre- to post-test for IL and CL (*p* < 0.001, d = 1.00), IL and CGw (*p* = 0.002, d = 0.97) and IL and CGs (*p* < 0.001, d = 0.92). There was an increase of 15.2% in KtW, while no significant increases could be obtained in the control conditions.

For ORTH, the two-way ANOVA revealed high effects for the time-dependent effect (η² = 0.11, *p* = 0.003) and time * group interactions (η² = 0.92, *p* < 0.001). The Scheffé test determined significant, large magnitude effect size increases from pre- to post-test for IL and CL (*p* < 0.001, d = 1.21), IL and CGw (*p* < 0.001, d = 1.46) and IL and CGs (*p* < 0.001, d = 1.49). However, no significant differences could be determined in the other groups with *p* = 0.66–0.99.

### 3.3. Intragroup Differences between Leg

Table 3 provides information for group effects between the strong and the weak leg in IG and CG and the ratio of imbalance (∆) as well as the level of significance. The *p*-values in the table provide information on whether there was a significant difference between the legs in the pairwise comparison between the legs within one group.

**Figure 4:**
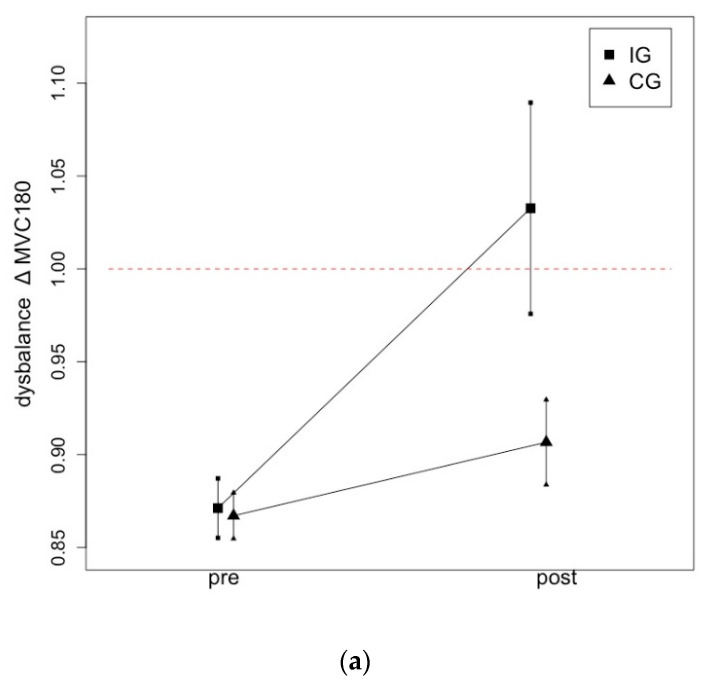

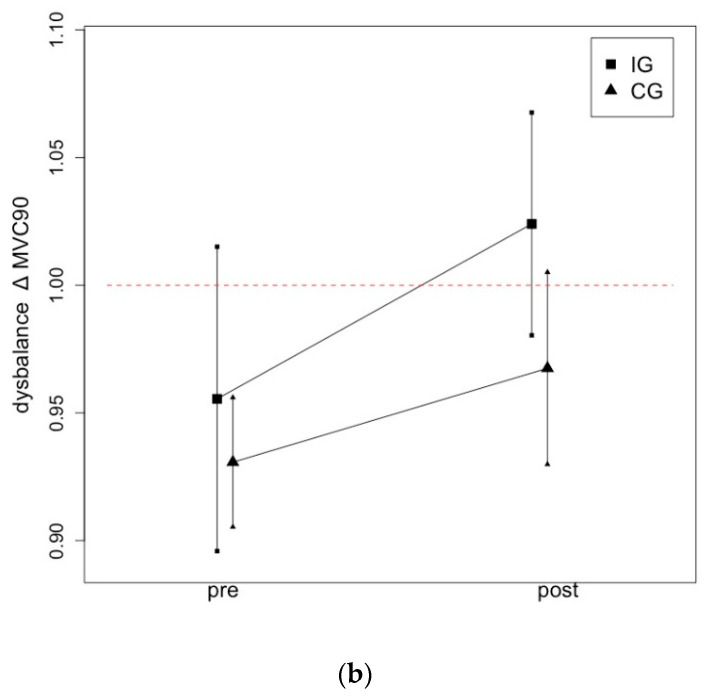
Graphical illustration of progression in ∆MSt in IG and CG following a one-hour daily stretching training of the plantar flexors in MVC180 (**a**) and MVC90 (**b**).

Figure 4 illustrates the changes from pre- to post-test in ∆MVC180 (a) and ∆MVC90 (b) following a one-hour daily static stretching intervention of the plantar flexors over six weeks.

**Figure 5:**
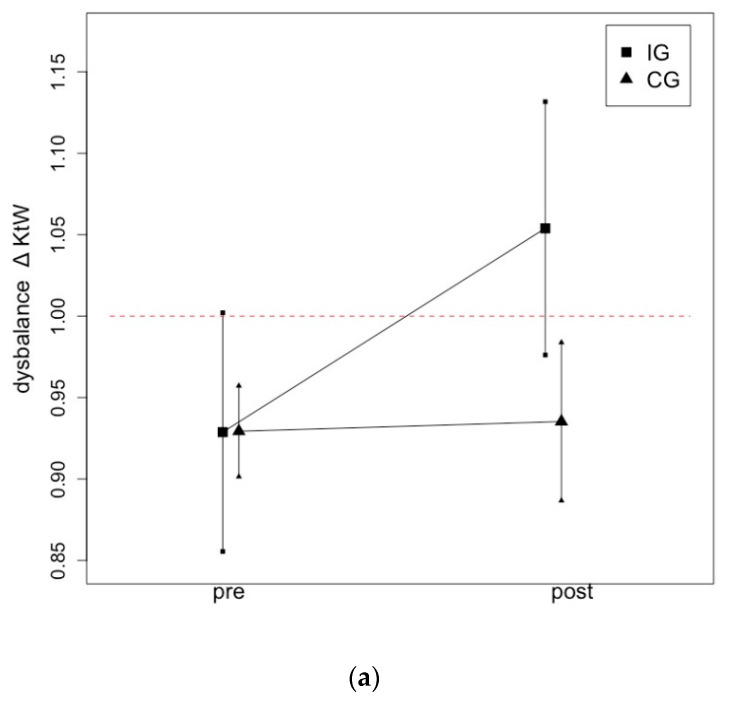

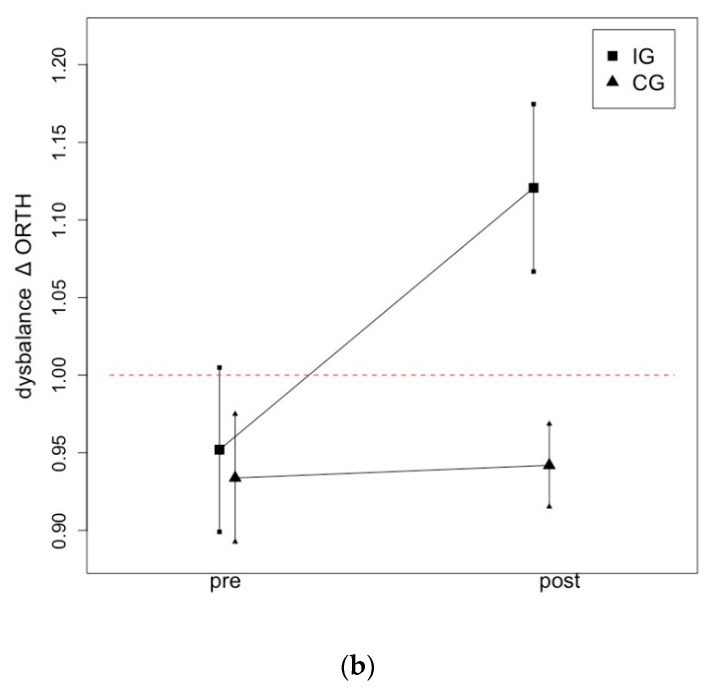
Graphical illustration of progression in ∆ROM in IG and CG following a one-hour daily stretching training of the plantar flexors on KtW (**a**) and ORTH (**b**).

Figure 5 illustrates the changes from pre- to post-test in ∆KtW (a) and ∆ORTH (b) due to a one-hour daily static stretching intervention of the plantar flexors over six weeks.

Post-hoc analysis for F-tests of G-Power calculated 1 − β = 76.8% for the lowest effect size with η² = 0.18, 1 − β = 99.99% for the highest effect size with η² = 0.39 with α = 0.05 for four groups and two time points for the time effect, 1 − β = 78.1% for the lowest effect size with η² = 0.22, and 1 − β = 100.00% for the highest effect size with η² = 0.55 with α = 0.05 for four groups and two time points for the interaction.

## 4. Discussion

The present study investigated the influence of a one-hour daily static stretching intervention of the calf muscles for six weeks on MSt and ROM in athletes who exhibited an MSt imbalance between their right and left plantar flexors before the start of the intervention. The results from the present study show that stretching the weak leg resulted in a full restoration of MSt and KtW in the plantar flexors due to a daily, one-hour stretching routine. In accordance with previous research [31], one-hour daily stretching training for the plantar flexors resulted in a significant moderate to large magnitude effect. The intervention induced such a degree of adaptation that it led to an imbalance in favor of the formerly weaker leg as the ROM increased more than 10% over the level of the previously stronger leg. As the results show post-test values of ∆ > 1 for MVC180, KtW and ORTH, the unilateral training intervention should be continually monitored to maintain an interlimb balance in strength and flexibility. 

In accordance with previous research [36], MSt in plantar flexors seems to be influenced by knee joint angle. Pre-test values showed a higher ∆ in MVC180, while in the same population, there was a lower ∆ for MVC90, indicating that an imbalance in MSt may be movement specific. Additionally, the improvements in MSt and ROM in the performance tests with an extended knee joint were higher compared to those tests with a flexed knee joint as the testing condition was more closely related to the training condition [37] and, therefore, indicating strong differences between testing characteristics. Nevertheless, long-duration static stretching seems to be a suitable method to increase MSt and ROM in the plantar flexors and, therefore, reduce imbalances if used in the weaker leg. In previous research, authors requested the application of long-duration static stretching in the rehabilitation of immobilization-induced atrophy and strength loss. 

Muscular imbalances can arise from focused unilateral performances, such as in golf [38] or tennis [39], but also from reduced activity due to injury, which can possibly be attributed to less tension because of pain or heightened neuromuscular inhibition (especially with eccentric contractions) [20,40] as well as complete immobilization after surgery, leading to significant atrophy and loss of function and strength [41]. Veldhuizen et al. [41] describe a 21 ± 7% decrease in MCSA and a 53 ± 9% decrease in peak torque after four weeks of immobilization, which often cannot be restored completely through common rehabilitation programs [12,42]. However, it must be considered that there is a high heterogeneity within rehabilitation programs, which further hinders comparisons [43,44]. Independent of the applied rehabilitation protocol, these strength deficits within the injured muscles can be detected up to 11 years after injury [16]. Similarly, limited voluntary quadriceps muscle activation was reported years after traumatic knee injuries [45]. This is of particular significance as it has been suggested that these prolonged deficits potentially increase the risk for recurrences due to persistent atrophy and (eccentric) strength deficits in the injured muscles [19,20]. Therefore, more physical exercise is required in the immediate post-injury period to regain lost muscle strength [17,46,47].

As Croisier [25] refers to a higher risk of injury due to an imbalance in strength capacity and flexibility in sport performance, the reduction in an imbalance in flexibility between legs is required. The present study showed that one hour of daily stretching resolved the flexibility deficit and led to improved flexibility compared with the previously more flexible leg. 

The first studies on this topic were conducted in the 1970–1990s in animal experiments, showing significant increases in MSt [48] and in muscle mass, MCSA and fiber length [29,49], leading to previous human studies showing the general transferability of results from animal experiments [31]. Based on this, it can be hypothesized that mechanical tension induced via stretching could be a sufficient stimulus to cause hypertrophy and, therefore, improve MSt [31]. It has been hypothesized that stretching can induce hypertrophy [50,51]. However, using short-duration stretching, the authors of [52] showed that this stimulus was not sufficient to produce significant hypertrophy with conflicting results regarding MSt. While authors showed significant enhancements of MSt after stretching durations of 4 × 30 s to 6 × 5 min [53,54,55], other authors were not able to induce increases in MSt by using stretching durations of up to 6 minutes [56,57]. Apostolopoulos et al. [58] and Thomas et al. [59] stated that a high impact of stretching duration and intensity, using long-duration stretching, can be hypothesized to be more effective compared to short-duration stretching. 

Previous studies investigating long-duration static stretching on MSt and ROM were performed to clarify the possibility of inducing MSt and ROM improvements due to static stretching and, therefore, examine the transferability of results from animals to humans. However, even if this study was not performed with patients in rehabilitation, it is the first study investigating those effects with a clear relationship to a relevant practical application setting. Therefore, it seems that the results could be of high impact in rehabilitation settings for patients without the ability or motivation to perform commonly used strength training. Consequently, further studies should address studies with patients after surgery, such as after anterior cruciate ligament ruptures, as the rehabilitation process takes a lot of time and often fails to reach the performance level from pre-surgery, which can also lead to a change in sport [8,42]. Since the present study provides no information about MCSA and MTh, further studies should include imaging of musculature to clarify potential hypertrophy, as a previous study showed that increases in MSt due to long-duration stretching were accompanied by enhanced muscle thickness. The usage of long-duration stretching interventions could possibly be an effective alternative to common strength training or rehabilitation exercise when there is no possibility for active force production due to restrictions on joint movement [60]. It could also provide supplementation of strength training to counteract immobilization-induced force deficits and muscle loss. From this, further studies should address this issue and examine the supplementation of long-duration stretching to rehabilitation routines or investigate the possibility of inducing stretching to reduce immobilization-induced atrophy within a short period after surgery, especially since early functional treatment of the muscle seems to be beneficial after surgery of, for example, Achilles tendon rupture repair, instead of immobilization [46,60]. 

### 4.1. Limitations

MSt was determined isometrically. However, Warneke et al. [36] stated serious differences in concordance between isometric and dynamic testing conditions. Because of limitations resulting from the high degree of angle specificity of isometric testing conditions and specific neuronal demands of isometric testing conditions, transferability to dynamic testing procedures must be questioned. Furthermore, only male participants were included in this study; however, the aim of this study was not to evaluate the influence of sex on the effectiveness of this training routine. Further studies should study these effects in female participants as well. Randomized assignment to the respective groups was not possible in this study design because not all subjects were willing to perform a one-hour daily stretching protocol. However, because there were no significant differences in pre-test scores, the results provide meaningful results in practice. Additionally, the post-hoc power test showed a power of 76–100%, showing that the sample size was sufficient to answer our research question adequately. It was not possible to include patients with previous phases of immobilization in this study; from this, further studies should address this population. Previous injuries and sports activities were not documented within the participants; therefore, the origins of imbalances cannot be reported but could possibly influence the magnitude of adaptation due to stretching. Therefore, it could be hypothesized that results are biased by different indications leading to the imbalance influencing the adaptational responses. Further research, especially in a clinical context, should, therefore, pay attention to this issue. In addition, because of the inclusion criteria of athletically active and healthy participants, the initial sample size was reduced to a large extent and might not be representative of the general population.

### 4.2. Practical Application

Malliares et al. [61] demonstrated the importance of well-trained dorsiflexion mobility in jumping performance in volleyball players to reduce the risk of patellar tendinopathy, while Keiner et al. [62] and Möck et al. [63] showed a moderate influence of MSt in the calf muscle on jumping performance and sprinting performance in elite sports. As there are high injury incidence rates of up to 17% in professional sports related to the ankle and thighs, there is a high relevance of the calf muscle and the ankle joint in athletic performance and should, therefore, be addressed in the prevention and rehabilitation. Wong and Hong [24] summarize that these lower-limb injuries entail time away from sport but also substantial medical expenses. Furthermore, several authors have requested effective, economic rehabilitation programs. The training intervention described in this study could be of high impact in the immediate post-operative mobilization and exercise process with a potential application as an alternative or supplementation to commonly used exercise programs. More research, especially addressing the optimal stretching duration, intervention period and training frequency, as well as the duration in which the effects remained when the intervention was stopped, is requested.

## 5. Conclusions

The results provide evidence for the application of long-duration (6 weeks of daily one-hour static stretching) static stretching in counteracting muscular strength and ROM imbalances in the plantar flexors. From this, further studies in a clinical setting are required to provide further evidence, investigate possible timepoints of usage after injury and determine the optimal stretching duration and frequency to counteract imbalances—and not to induce new imbalances.

## Figures and Tables

**Table 1:** Values for the reliability of the included tests.

Parameter	ICC (95% CI)	CV (95% CI)
MVC180	0.996 (0.993–0.998)	1.81% (1.43–2.23)
MVC90	0.957 (0.920–0.977)	3.61% (1.88–6.11)
KtW	0.975 (0.953–0.987)	3.54% (2.47–4.83)
ORTH	0.982 (0.978–0.986)	2.59% (2.11–3.01)

MVC180 = maximal voluntary contraction in the plantar flexors with extended knee joint, MVC90 = maximal voluntary contraction in the plantar flexors with bent knee joint, KtW = ROM measured via knee-to-wall stretch testing device, ORTH = ROM testing via angle measuring device of the orthosis.

**Table 2:** Descriptive statistics and results of two-way ANOVA considering both legs of both groups. The Time Effect, as well as the Time * Group effect, are provided in the table.

Parameter	Pre-test (M ± SD)	Post-test (M ± SD)	Pre-Post Diff in %	Time Effect	Time * Group
MVC180IL	1660.87 ± 349.05	1982.67 ± 409.20	+19.9 ± 11.1	*p* < 0.001 F_1,54_ = 34.98 η² = 0.39	*p* < 0.001 F_3,54_ = 22.19 η² = 0.55
MVC180CL	1909.93 ± 413.17	1933.40 ± 403.61	+1.5 ± 6.4		
MVC180w MVC180s	1499.29 ± 331.46 1728.71 ± 377.56	1556.79 ± 338.13 1713.71 ± 342.41	+3.9 ± 4.2 −0.5 ± 4.0		
MVC90IL	1435.73 ± 340.85	1550.33 ± 324.96	+9.6 ± 9.9	*p* < 0.001 F_1,54_ = 12.14 η² = 0.18	*p* = 0.004 F_3,54_ = 5.05 η² = 0.22
MVC90CL	1488.67 ± 279.28	1518.40 ± 316.38	+1.7 ± 6.9		
MVC90w MVC90s	1342.36 ± 238.04 1441.36 ± 237.02	1376.00 ± 206.75 1428.93 ± 235.96	+3.0 ± 5.6 −0.7 ± 5.7		
KtWIL	12.13 ± 2.51	13.87 ± 2.35	+15.2 ± 7.6	*p* < 0.001 F_1,54_ = 20.66 η² = 0.28	*p* < 0.001 F_3,54_ = 5.52 η² = 0.349
KtWCL	13.1 ± 1.97	13.23 ± 1.82	+1.3 ± 5.6		
KtWCGw KtWCGs	11.39 ± 2.44 12.18 ± 2.12	11.57 ± 2.15 12.43 ± 1.59	+2.9 ± 10.1 +4.2 ± 18.9		
ORTHIL	7.9 ± 2.32	9.43 ± 2.04	+22.4 ± 15.5	*p* < 0.001 F_1,54_ = 24.12 η² = 0.31	*p* < 0.001 F_3,54_ = 20.47 η² = 0.53
ORTHCL	8.30 ± 2.28	8.53 ± 2.23	+3.2 ± 5.7		
ORTHCGw ORTHs	7.75 ± 1.25 8.29 ± 1.07	7.71 ± 1.01 8.21 ± 1.17	0.7 ± 11.5 −0.9 ± 5.0		

MVC180 = maximal voluntary contraction in the plantar flexors with extended knee joint, MVC90 = maximal voluntary contraction in the plantar flexors with bent knee joint, KtW = ROM measured via knee-to-wall stretch testing device, ORTH = ROM testing via angle measuring device of the orthosis, IL = intervened leg (IG), CL = control leg (IG), w = weak leg (CG), s = strong leg (CG).

**Table 3:** Group effects between the strong and the weak leg in IG and CG.

Parameter.	IG		CG	
	Pre-test	Post-test	Pre-test	Post-test
MVC180	**∆= 0.87** *p* < 0.001	**∆= 1.03** *p* = 0.19	**∆= 0.87** *p* < 0.001	**∆= 0.91** *p* < 0.001
MVC90	**∆= 0.96** *p* = 0.92	**∆= 1.02** *p* = 0.17	**∆= 0.93** *p* < 0.001	**∆= 0.96** *p* = 0.045
KtW	**∆= 0.92** *p* = 0.029	**∆= 1.05** *p* = 0.11	**∆= 0.93** *p* < 0.001	**∆= 0.93** *p* = 0.018
ORTH	**∆= 0.95** *p* = 0.027	**∆= 1.11** *p* < 0.001	**∆= 0.93** *p* = 0.004	**∆= 0.94** *p* < 0.001

MVC180 = maximal voluntary contraction in the plantar flexors with extended knee joint, MVC90 = maximal voluntary contraction in the plantar flexors with bent knee joint, KtW = ROM measured via knee-to-wall stretch testing device, ORTH = ROM testing via angle measuring device of the orthosis.

## Data Availability

Data are available upon reasonable request.
